# General Anesthesia for Patients With Chronic Obstructive Pulmonary Disease and Postoperative Respiratory Failure: A Retrospective Analysis of 120 Patients

**DOI:** 10.3389/fphys.2022.842784

**Published:** 2022-05-30

**Authors:** Ruixue Hou, Fangfang Miao, Di Jin, Qingfang Duan, Cheng Yin, Qunpeng Feng, Tianlong Wang

**Affiliations:** Department of Anesthesiology, Xuanwu Hospital, Capital Medical University, Beijing, China

**Keywords:** COPD, general anesthesia, respiratory failure, spinal surgery, lung function test

## Abstract

**Background:** Chronic obstructive pulmonary disease (COPD) has been considered a risk factor for postoperative respiratory failure after general anesthesia. However, the association between COPD severity and postoperative respiratory failure among COPD patients is unknown. Our aim was to compare the prevalence of postoperative respiratory failure in COPD patients according to disease severity after general anesthesia.

**Methods:** We retrospectively reviewed COPD patients undergoing spinal surgery with general anesthesia at our clinical center between January 2016 and January 2021. These subjects were divided into four groups (group I = mild COPD, group II = moderate COPD, group III = severe COPD, and group IV = very severe COPD) according to their preoperative lung function. The primary endpoint was a respiratory failure 1 week after surgery. The diagnosis of respiratory failure was made with the presence of one or more of the following criteria: prolonged ventilator dependence, unplanned postoperative intubation, and partial pressure of arterial oxygen (PaO_2_) ≤ 50 mmHg while the patient was breathing ambient air in the hospital. The extubation time, perioperative PaO_2_ and partial pressure of arterial carbon dioxide (PaCO_2_), postoperative lung infection, and length of hospitalization were also compared.

**Results:** A total of 120 patients who underwent spinal surgery with general anesthesia were included in this retrospective study. Postoperative respiratory failure occurred in 0 (0.0%) patient in group I, 1 (1.5%) patient in group II, 1 (2.5%) patient in group III, and 1 (14.5%) patient in group IV 1 week after surgery (*p* = 0.219). The duration of anesthesia was 243.3 ± 104.3 min in group I, 235.5 ± 78.8 min in group II, 196.0 ± 66.3 min in group III, and 173.1 ± 63.7 min in group IV (*p <* 0.001). Preoperative PaO_2_, PaCO_2_, intraoperative oxygenation index [a ratio of PaO_2_ to fraction of inspired oxygen (FiO_2_)], and postoperative PaO_2_ were significantly different among the four groups (*p* < 0.001, 0.001, 0.046, <0.001, respectively). No significant differences among the four groups were seen in extubation time, pulmonary infection, or hospital stay (*p* = 0.174, 0.843, 0.253, respectively). The univariate analysis revealed that higher preoperative PaO_2_ was associated with a lower rate of postoperative respiratory failure (OR 0.83; 95% CI, 0.72 to 0.95; *p* = 0.007).

**Conclusion:** The severity of COPD as assessed with GOLD classification was not associated with the development of postoperative respiratory failure. However, lower preoperative PaO_2_ was associated with greater odds of postoperative respiratory failure in COPD patients.

## Introduction

Chronic obstructive pulmonary disease (COPD) is a risk factor for postoperative morbidity and mortality (Nafiu et al., 2011; Gupta et al., 2013), and it is associated with prolonged mechanical ventilation after surgery (Licker et al., 2006). Prolonged mechanical ventilation can result in ventilator-induced lung injury and pulmonary infection, which can exacerbate pulmonary function in these high-risk patients (Beitler et al., 2016; Gattinoni et al., 2016). At the same time, delayed weaning is associated with increased hospital stay and treatment costs (Ball et al., 2018; Kocyigit et al., 2021). This places a strain on both surgeons and anesthesiologists (Halbert et al., 2006). Given the circumstances, early extubation is advised. However, clinicians must assess the risk of extubation failure because reintubation increases the risk of hospital-acquired pneumonia by eight times and death by six-twelve times (Liang et al., 2012). It is critical to determine the best time for COPD patients to be extubated after surgery.

According to the Global Initiative for Chronic Obstructive Lung Disease (GOLD) standard (Vogelmeier et al., 2017), COPD can be classified into four stages (mild, moderate, severe, very severe) based on the ratio of forced expiratory volume in 1 second to normal predicted (FEV_1_% pred). Several preclinical studies and meta-analyses have concluded that epidural or spinal anesthesia is preferable to general anesthesia for COPD patients in order to reduce perioperative complications and prolonged mechanical ventilation (Upchurch et al., 2003; Hausman et al., 2015; Bayrak and Altıntas, 2018). However, due to abnormal coagulation or surgical sites, general anesthesia may be the only option for these patients in some cases (Gulur et al., 2015). Should this be considered a contraindication to selective surgical procedures and general anesthesia, or should surgery be postponed for these high-risk patients? Furthermore, should clinical treatment decisions be individualized to the severity of COPD? We must weigh the risks of general anesthesia against the risk of postoperative complications and patient delays or cancellations. We should not deny patients access to surgical treatments solely because of their comorbidity.

In this observational study, our main objective was to explore the relationship between the severity of COPD patients who underwent spinal surgery and the prevalence of postoperative respiratory failure. Additionally, various candidate risk factors were analyzed to determine whether they were good predictors of postoperative respiratory failure.

## Methods

The ethical review board of the Capital Medical University Xuanwu Hospital approved this study (ChiCTR2100049597) and waived the need for informed consent. From January 2016 to January 2021, 120 patients with COPD who had underwent spinal surgery at the Capital Medical University Xuanwu Hospital were included in this study. The data from the Electronic Medical Record were extracted, including demographics, spirometry results, blood gas analysis, comorbidities, postoperative outcomes up to 30 days, and other variables. Patients who required emergent surgery lacked pulmonary function reports, or had other severe organ-system diseases (e.g., cardiovascular and/or neurological illnesses, hepatic and/or kidney dysfunction) were excluded.

The diagnosis of COPD was confirmed by a ratio of FEV_1_ to forced vital capacity (FVC) less than 70% after inhaling a bronchodilator. COPD patients were divided into four groups according to the severity defined by spirometry tests on the basis of GOLD guidelines (Vogelmeier et al., 2017). Mild COPD was defined as FEV_1_ ≥ 80% of the predicted value, moderate COPD was defined as 50% ≤ FEV_1_ < 80% of the predicted value, severe COPD was defined as 30% ≤ FEV_1_ < 50% of the predicted value, and very severe COPD was defined as FEV_1_ < 30% of the predicted value.

The primary outcome was a respiratory failure 1 week after surgery. Respiratory failure was defined as the presence of one or more of the following criteria: prolonged ventilator dependence, unplanned postoperative intubation, or PaO_2_ ≤ 50 mmHg while the patient was breathing ambient air in hospital (Attaallah et al., 2019). Additionally, the extubation time, perioperative PaO_2_ and PaCO_2_, postoperative lung infection, and length of hospitalization were reviewed.

### Statistical Analysis

All statistical analyses were performed using SPSS 19.0 software. Continuous variables were analyzed with Student’s t test (normal distribution), the Wilcoxon rank test (skewed distribution), or analysis of variance (ANOVA). Categorical data were analyzed using the chi-squared test or Fisher’s exact test. Risk factors for postoperative respiratory failure were identified in a literature review (Attaallah et al., 2019). Univariate analyses were conducted to examine the association between previously potential risk factors (including age, sex, BMI, hypertension, diabetes mellitus, smoking history, ASA score, length of anesthesia, perioperative arterial blood gas, and spirometry findings) and postoperative respiratory failure. Analyses were considered statistically significant at *p* < 0.05.

## Results

**FIGURE 1:**
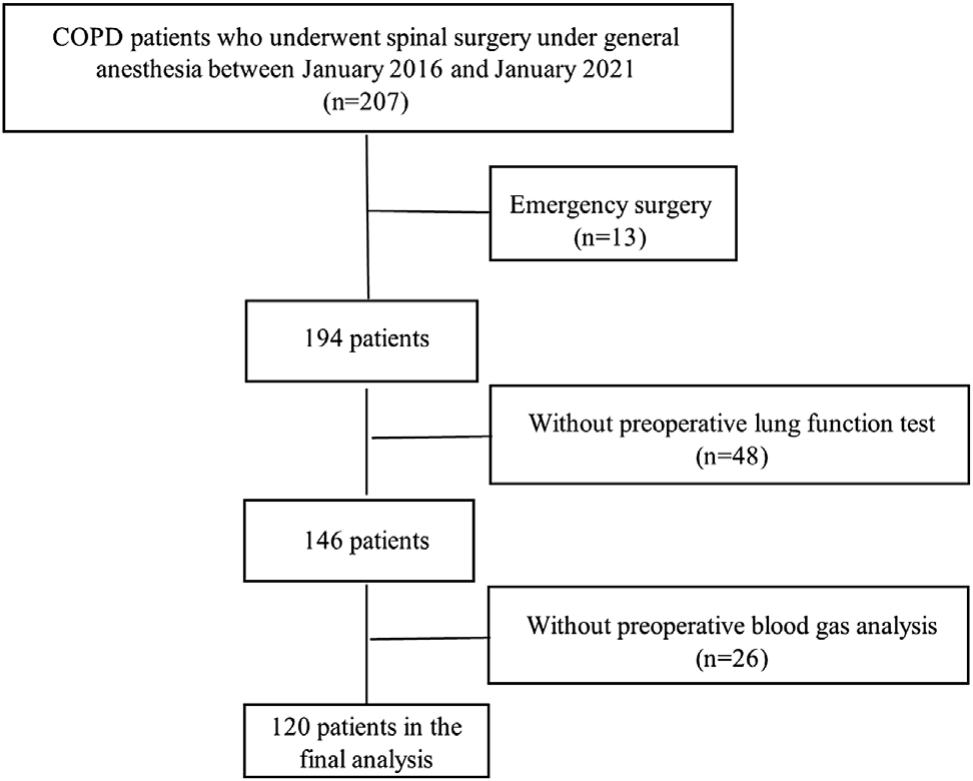
Flow diagram for patients.

Between January 2016 and January 2021, 207 patients were screened for study participation; of these, 120 patients were included in the final analysis of the study; the remaining 87 were excluded from the study due to emergent surgery (*n* = 13), unavailable preoperative lung function test (*n* = 48), or lack of preoperative blood gas analysis (*n* = 26) (Figure 1). These patients were divided into four groups according to the value of FEV_1_ of the predicted value: group I (*n* = 7, FEV_1_% pred 81.9 ± 2.1), group II (*n* = 66, FEV_1_% pred 62.9 ± 8.1), group III (n = 40, FEV_1_% pred 43.6 ± 6.5), and group IV (*n* = 7, FEV_1_% pred 25.9 ± 3.0). The preoperative FEV_1_ of the predicted value was not associated with postoperative respiratory failure in the univariate analyses (Table 3). The duration of anesthesia was 243.3 ± 104.3 min in group I, 235.5 ± 78.8 min in group II, 196.0 ± 66.3 min in group III, and 173.1 ± 63.7 min in group IV (*p* = 0.021). Baseline characteristics including demographic data, ASA class, New York Heart Association (NYHA) class, and prevalence of individual comorbidities (hypertension, diabetes, coronary artery disease, history of stroke) were generally comparable among the four groups (Table 1).

**TABLE 1:** Demographics and intraoperative variables.

	I (*n* = 7)	II (*n* = 66)	III (*n* = 40)	IV (*n* = 7)	*p*-value
FEV_1_ (% pred)	81.9 ± 2.1	62.9 ± 8.1	43.6 ± 6.5	25.9 ± 3.0	<0.001
Age (yr)	63.9 ± 9.8	72.9 ± 9.4	71.5 ± 9.4	68.3 ± 9.9	0.083
Male, No (%)	5 (71.4)	46 (69.7)	24 (60.0)	7 (100.0)	0.202
BMI (kg/m^2^)	24.8 ± 4.2	24.3 ± 4.8	22.6 ± 7.1	23.4 ± 3.3	0.502
Current smoking, No (%)	3 (42.9)	15 (23.1)	5 (12.5)	2 (28.6)	0.244
Comorbidities, No (%)					
Hypertension	6 (85.7)	47 (74.6)	24 (70.6)	5 (71.4)	0.864
Diabetes	2 (28.6)	14 (21.5)	9 (23.1)	1 (14.3)	0.929
Coronary artery disease	1 (14.3)	14 (21.2)	13 (32.5)	1 (14.3)	0.462
History of stroke	1 (14.3)	18 (27.3)	5 (12.5)	0 (0.0)	0.140
Type of surgery, No. (%)					0.350
Cervical surgery	4 (57.1)	30 (45.5)	24 (60.0)	5 (71.4)	
Lumbar surgery	3 (42.9)	36 (54.5)	16 (40.0)	2 (28.6)	
ASA class, No (%)					0.883
I	0 (0.0)	1 (1.5)	0 (0.0)	0 (0.0)	
II	2 (28.6)	25 (37.9)	15 (37.5)	3 (42.9)	
III	5 (71.4)	38 (57.6)	24 (60.0)	3 (42.9)	
IV	0 (0.0)	2 (3.0)	1 (2.5)	1 (14.3)	
NYHA class, No. (%)					0.418
I	2 (28.6)	16 (24.2)	7 (17.5)	2 (28.6)	
II	4 (57.1)	42 (63.6)	22 (55.0)	5 (71.4)	
III	1 (14.3)	8 (12.1)	11 (27.5)	0 (0.0)	
Duration of anesthesia (min)	243.3 ± 104.3	235.5 ± 78.8	196.0 ± 66.3	173.1 ± 63.7	0.021

FEV1, forced expiratory volume in 1s; ASA, American society of anesthesiologists; BMI, body mass index; NYHA, New York heart association.

**FIGURE 2:**
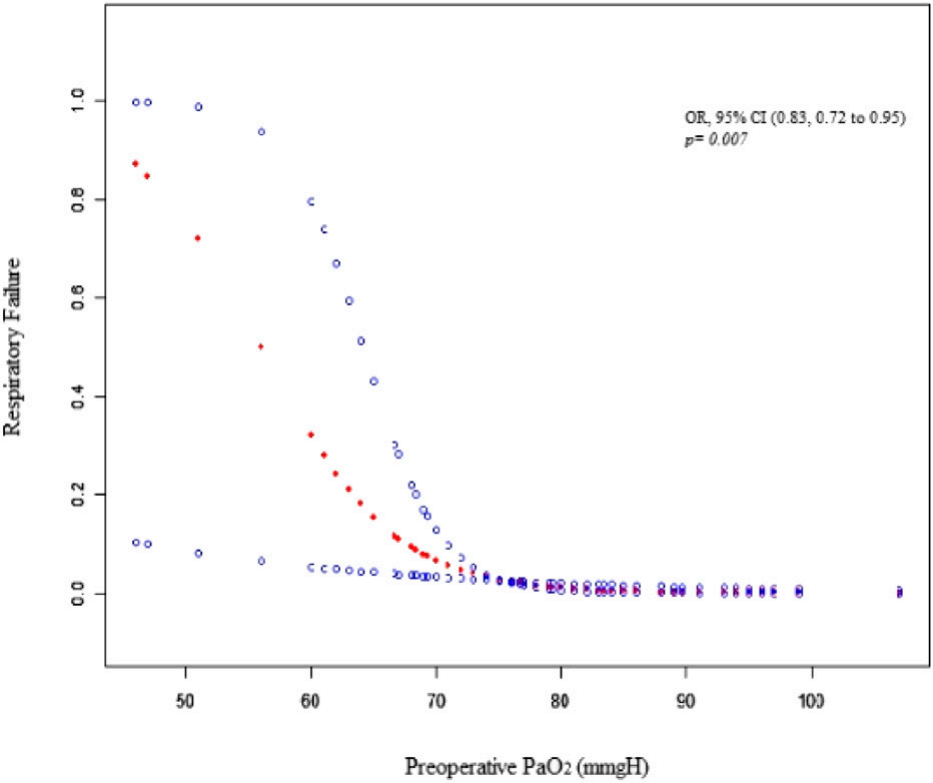
Association of preoperative PaO_2_ with respiratory failure.

Perioperative blood gas analyses are shown in Table 2. Preoperative PaO_2_, PaCO_2_, intraoperative oxygenation index and postoperative PaO_2_ were significantly different among the four groups (*p* < 0.001, 0.001, 0.046, <0.001, respectively). In the univariate analyses, preoperative PaCO_2_, intraoperative oxygenation index, and the duration of anesthesia were not associated with postoperative respiratory failure (*p* = 0.454, 0.107, 0.302, and 0.246, respectively) (Table 3). However, higher preoperative PaO_2_ was associated with a lower rate of postoperative respiratory failure (OR 0.83; 95% CI, 0.72 to 0.95; *p* = 0.007) (Figure 2). We further applied a two-piecewise linear regression model to examine the threshold effect of the preoperative PaO_2_ using a smoothing function (Figure 2). We found that a preoperative PaO_2_ (room air) of less than 68 mmgH was associated with the probability of postoperative respiratory failure.

**TABLE 2:** Perioperative blood gas analysis.

		I (n = 7)	II (n = 66)	III (n = 40)	IV (n = 7)	*p*-value
Preoperative	PaO_2_ (mmHg)	84.4 ± 8.2	77.2 ± 10.6	72.7 ± 8.9	63.3 ± 12.3	<0.001
	PaCO_2_ (mmHg)	38.1 ± 4.2	38.5 ± 3.3	41.6 ± 4.4	39.9 ± 5.3	0.001
Intraoperative	PaO_2_ (mmHg)	210.6 ± 31.8	197.7 ± 71.5	187.8 ± 48.7	186.0 ± 45.7	0.726
	PaCO_2_ (mmHg)	41.9 ± 2.9	41.5 ± 3.7	43.0 ± 4.0	42.9 ± 3.8	0.269
	Oxygenation index	434.6 ± 63.0	387.2 ± 93.7	370.2 ± 97.0	300.2 ± 82.7	0.046
After extubation	PaO_2_ (mmHg)	170.4 ± 28.0	113.5 ± 30.2	107.0 ± 42.0	85.7 ± 23.0	<0.001
	PaCO_2_ (mmHg)	43.4 ± 1.6	44.4 ± 4.2	46.0 ± 5.3	47.4 ± 5.8	0.135

PaO2, partial pressure of arterial oxygen; PaCO2, partial pressure of arterial carbon dioxide; oxygenation index, a ratio of PaO2 to a fraction of inspired oxygen.

**TABLE 3:** Risk factors for respiratory failure.

	OR (95% CI)	*p*-value
Preoperative PaO_2_ (mmHg)	0.83 (0.72–0.95)	0.007
Preoperative PaCO_2_ (mmHg)	1.1 (0.85–1.43)	0.454
FEV_1_ (% pred)	0.93 (0.85–1.02)	0.107
Oxygenation index	0.99 (0.98–1)	0.302
Duration of anesthesia	0.99 (0.97–1.01)	0.246

PaO_2_, partial pressure of arterial oxygen; PaCO_2_, partial pressure of arterial carbon dioxide; FEV_1_ (% pred), a ratio of forced expiratory volume in 1 s to normal predicted; oxygenation index: a ratio of PaO_2_ to a fraction of inspired oxygen.

Postoperative respiratory failure occurred in 1 (1.5%) patient in group II, 1 (2.5%) patient in group III, and 1 (14.5%) patient in group IV 1 week after surgery (*p* = 0.219). The percentage of patients who needed mechanical ventilation after surgery was also similar (*p* = 0.497). No other significant differences among the four groups were observed in extubation time, pulmonary infection, or length of hospital stay after surgery (Table 4).

**TABLE 4:** Postoperative pulmonary complications.

	I (*n* = 7)	II (*n* = 66)	III (*n* = 40)	IV (*n* = 7)	*p*-value
Extubation time (min)	8.1 ± 2.0	9.5 ± 1.9	9.4 ± 2.2	10.8 ± 4.1	0.174
Patients with endotracheal intubation on ICU admission after surgery	0 (0.0%)	2 (3.0%)	1 (5.%)	1 (14.3%)	0.497
Respiratory failure	0 (0.0%)	1 (1.5%)	1 (2.5%)	1 (14.5%)	0.219
Pulmonary infection	0 (0.0%)	1 (1.5%)	0 (0.0%)	0 (0.0%)	0.843
Length of hospital stay (d)	11.00 ± 2.2	13.0 ± 4.6	11.7 ± 2.7	12.9 ± 2.0	0.253

## Discussion

The current study indicated that patients with different severity of COPD, defined as FEV_1_%pred, were not related to postoperative respiratory failure. By analyzing the potential risk factors before surgery, we found that lower PaO_2_ resulted in a higher rate of respiratory failure after surgery among COPD patients (OR 0.83; 95% CI, 0.72–0.95; *p* = 0.007). To the best of our knowledge, this is the first study to explore whether the severity of COPD is associated with postoperative respiratory failure undergoing spinal surgery.

Pulmonary function testing is often used to diagnose and evaluate patients’ pulmonary disease (Wang, 2004). Its good quality is influenced by a variety of factors, including subjects’ cooperation, sophisticated technologist, and precise instrumentation (Culver et al., 2017). Sotirios (Kakavas et al., 2021) et al. believed that the calculation of the FEV_1_ alone has limitations in detecting the underlying complexity of COPD disease. Our study also found that compared to the pulmonary function test, preoperative PaO_2_ is a more objective and reliable parameter to evaluate the risk of postoperative respiratory failure in COPD patients. Preoperative PaO_2_ (room air) of less than 68 mmgH was associated with the probability of postoperative respiratory failure.

Many factors can increase the rate of postoperative pulmonary complications (Sabaté et al., 2014; Canet et al., 2015; Yang et al., 2015). COPD is an independent factor after general anesthesia (Manganas et al., 2007; Jonker et al., 2009; Xiao et al., 2020). In the current study, the demographic and preoperative comorbidities were equally distributed, and patients undergoing emergency surgery were excluded. Thus, we expected to exclude the potential bias caused by a mismatch among groups. Fields and Divino’s (2016) study reported that patients with COPD undergoing abdominal procedures had increased morbidity and duration of stay. Unfortunately, they did not analyze the effect of the severity of COPD on postoperative outcomes. No significant differences among the four groups were observed in pulmonary infection or the length of hospital stay after surgery in our study. Such a difference might be attributed to differences in a variety of factors, including surgery type, the duration of surgery, and patient demographic features. Upper abdominal surgery plays an important role in postoperative diaphragmatic dysfunction, a well-known cause of postoperative pulmonary complications (Berdah et al., 2002; Kim et al., 2016). The duration of anesthesia was shorter in patients in group IV than in the other groups; however, neither was an independent predictor for postoperative respiratory failure in the univariate analysis. We speculated that surgeons chose minimally invasive surgery as much as possible to shorten the operation time and reduce postoperative complications.

The present study has several limitations that should be noted. First, this was a retrospective analysis performed at a single center. We were unable to get access to the databases about the intraoperative ventilation strategy and postoperative conditions such as pain and inflammation and which patient used NIV or HFNC after the operation, which may affect outcomes (D'Souza et al., 2020). Second, in the present study, we found that preoperative PaO_2_ provides a means of objectively evaluating the fitness and can be used to suggest individualized risk stratification in predicting postoperative pulmonary complications. However, the sample size is relatively small which may have affected the statistical power of the comparisons. Third, the blood gas analysis after extubation is not comparable because the test time and inhaled oxygen concentration were different after extubation. Well-designed prospective studies are necessary to address these issues.

## Conclusion

Patients with different severities of COPD defined as FEV_1_%pred were not related to postoperative respiratory failure. However, a lower preoperative PaO_2_ was associated with greater odds of postoperative respiratory failure in COPD patients.

## Data Availability

The raw data supporting the conclusion of this article will be made available by the authors, without undue reservation.
